# Aerobic and Anaerobic Metabolism During Monofin Swimming in Trained Breath-Hold Divers

**DOI:** 10.3390/jfmk10020218

**Published:** 2025-06-06

**Authors:** Ivan Drviš, Dario Vrdoljak, Goran Dujić, Nikola Foretić, Željko Dujić

**Affiliations:** 1 Faculty of Kinesiology, University of Zagreb, 10000 Zagreb, Croatia; ivan.drvis@kifst.unizg.hr; 2 Faculty of Kinesiology, University of Split, 21000 Split, Croatia; darvrd@kifst.hr (D.V.); nikola.foretic@kifst.eu (N.F.); 3 Clinical Department of Diagnostic and Interventional Radiology, University Hospital of Split, 21000 Split, Croatia; goran.dujic@mefst.hr; 4 High Performance Sport Center, Croatian Olympic Committee, 10000 Zagreb, Croatia; 5 Department of Integrative Physiology, School of Medicine, University of Split, 21000 Split, Croatia

**Keywords:** freediving, dynamic, oxygen saturation, lactates, apnea

## Abstract

**Background:** This study aimed to examine the difference in blood lactate and oxygen saturation between monofin swimming with respiration and with breath-hold diving. The second aim was to investigate the difference between elite and intermediate breath hold-divers. **Methods:** This study included 15 freediving athletes (five females). Their chronological age was 25.9 ± 2.9 years, body mass 75.5 ± 11.9 kg, and height 180.2 ± 8.9 cm. The sample of variables included anthropometric indices, blood lactate, and oxygen saturation. The participants were measured during 100 m monofin horizontal swimming with respiration and breath-hold diving. Descriptive and inferential statistics were measured. The Kolmogorov–Smirnov (K-S) test was used to determine the normality of distribution. To determine the differences between the groups of participants, the *t*-test was used to determine the differences in anthropometric indices. Furthermore, to observe the differences between repeated measures, ANOVA with Fischer LSD test was used. Following that, the two-factor ANOVA analysis group (respiration/apnea) and group (intermediate/elite level)) was performed to determine the possible differences between groups in both conditions. Also, Cohen’s effect size was calculated to quantify the differences among the measurements. **Results:** The results show that the divers perceive significantly smaller levels of lactates during dives with respiration (intermediate, 2.44 ± 0.64 mmol/L; elite, 2.23 ± 0.34 mmol/L) than during apnea (intermediate, 6.06 ± 2.00 mmol/L; elite, 4.10 ± 0.66 mmol/L). Furthermore, intermediate freedivers tend to perceive significantly higher values. **Conclusions:** To conclude, it can be noted that apnea monofin diving elicits significantly higher lactate production in comparison with distance-matched swimming. Such findings imply the anaerobic nature of breath-holding diving. Apart from that, elite divers tend to show a lower accumulation of lactate. Such findings imply that elite-level divers can endure prolonged apneas with lower anaerobic metabolism use.

## 1. Introduction

Breath-hold diving (BHD) is a unique sport in which athletes hold their breath during a dive. Elite divers achieve results of up to 10 min and achieve a depth of up to 200 m [1,2]. To achieve such impressive results, the human body experiences multiple physiological responses. Firstly, both the sympathetic and parasympathetic nervous systems are activated during apneas [3,4]. To be precise, increased vagal activity activates apneic bradycardia (i.e., decrease in heart rate), whereas peripheral vasoconstriction is induced by a sympathetic activation [4,5]. Secondly, as a result of parasympathetic and sympathetic activity BHD athletes experience a reduction in cardiac output, with an increase in blood pressure [6,7]. Collectively, all of the above responses lead to a phenomenon called the human diving response (DR) as an adaptation to intentional and unintentional apneas [8]. During exercise, the diving response is strong enough to compensate for exercise-induced tachycardia during the apnea phase [9,10,11]. Due mostly to bradycardia, the cardiac output is decreased during exercise apneas, while the systemic vascular resistance rises [12].

These statements lead to the conclusion that success in competitive apnea is determined by both anaerobic and aerobic capacity [13]. Therefore, apnoeic diving capacity is pre-determined by the total aerobic and anaerobic metabolic stores, the asphyxia tolerance, and the rate at which the resources are used, i.e., metabolic rate [13,14]. Therefore, aerobic capacity plays a significant role in performance. Additionally, during BHD oxygen is used from stores in the blood, and other tissues [15]. Since there is a limited amount of oxygen blood flow is directed mainly toward the brain and heart, while the rest of the organism receives a restricted blood flow [16,17]. Hence, a drop in arterial oxygen saturation while breath-holding indicates an aerobic influence in divers, since divers use their stored oxygen during the dives.

Since freedivers rely on a single breath, their oxygen reserves are limited. Also, restriction to blood flow and reduced cardiac output limit the circulation of oxygen during BHD [18,19,20]. This condition, together with an increase in arterial pressure of carbon dioxide and decrease in arterial pressure of oxygen [15], leads to high activation of the anaerobic system. Apart from this, during dynamic apnea, working muscles receive reduced blood flow, which leads to relatively high lactate accumulation [21]. Following, both static (SA) and dynamic apnea (DA), demonstrate lactate accumulation and hypercapnia. As mentioned above, selective vasoconstriction during apnea, lactate removal from working muscles, and its transport could be impaired, resulting in limited oxidation, which may lead to its accumulation [13,14,21,22,23]. Such states influence and limit a diver’s ability to perform at the highest level.

As mentioned above, both aerobic and anaerobic capacities are important factors in diving performance. However, the literature review showed sparse evidence of these capacities during situational breath-hold diving. Regarding this, studies showed that lactic acid accumulation and hypercapnia during apnea may lead to severe acidosis, which can limit performance [24], and that oxygen preservation leads to a low metabolic rate, which can postpone the so-called “breaking point” of apnea and point to hypoxic loss of consciousness [8,24]. Primarily, this study aimed to examine the difference between monofin diving with respiration and with breath-hold in blood lactate accumulation and arterial oxygen saturation. Additionally, the second aim was to investigate the difference between elite and intermediate breath hold-divers in the mentioned parameters.

## 2. Materials and Methods

### 2.1. Participants

The sample of participants included 15 freediving athletes (5 females). Their chronological age was 25.9 ± 2.9 years, body mass 75.5 ± 11.9 kg, and height 180.2 ± 8.9 cm. Participants’ training experience was 3.5 ± 1.9 years. The sample included athletes who compete on a national and also on an international level. Six of them were national champions and national and world record holders; therefore, they are considered elite-level athletes. The other nine were national team athletes who competed in national and international competitions; they are considered intermediate freedivers. Participants did not have any illness or medical condition that might have prevented them from performing tests. They were informed about the procedures and purpose of the study and signed informed consent before the investigation began. The study was conducted following the declaration of Helsinki and the Ethical Board of the Faculty of Kinesiology, University of Split, Croatia (ethical board number 2181-205-02-05-22-035).

### 2.2. Variables

The variables in this study included anthropometric indices, blood lactate, and arterial oxygen saturation measurements.

Anthropometric indices included body height and body mass. Body mass was assessed with the bioimpedance scale (Tanita BC 418 scale; Tokyo, Japan). Whereas body height was measured with a measuring tape.

Apart from anthropometric indices, blood lactates (2 min after), and arterial oxygen saturation (immediately after) were measured. Blood lactates (b[LA]) were measured from the fingertip with a lactate analyzer (h/p/Cosmos Sirius, Leipzig, Germany). Arterial oxygen saturation (SaO_2_) was assessed from the finger with an oximeter Edan H100 (Edan Instruments Inc., Shenzhen, China). Both b[LA] and SpO_2_ were measured before the start of the test to determine the baseline values. They were also measured immediately after both tests (100 m with respiration and 100 m apnea). The monofin swimming test was used, as it was the main discipline of measured participants.

### 2.3. Procedures

**Figure 1:**
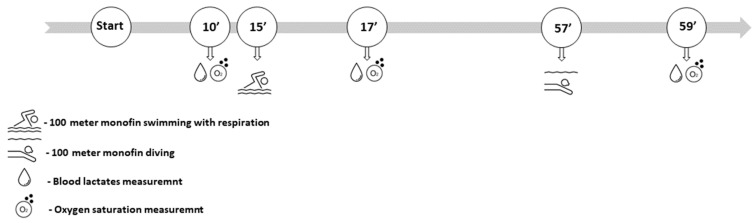
Data collection timeline: before the tests and directly after 100 m respiration and 100 m apnea test.

The procedure was performed in the 25 m swimming pool in the morning hours. Also, the procedure was performed after the competition season had ended to provide the most stable results and the highest performance. Upon arrival, participants rested for 10 min for a better assessment of b[LA]. In addition, baseline SpO_2_ was determined during that period. Afterward, participants executed a 100 m monofin swim with normal respiration. They were equipped with a monofin and diving snorkel to allow divers to breathe. After they finished the 100 m monofin respiration test, they took a 40 min rest to allow themselves a full recovery. The second test was conducted in the same manner apart from respiration. A 100 m monofin apnea was performed with the head of the participant submerged underwater, to prevent respiration. After both tests, both b[LA] and SpO_2_ were assessed. Both with respiration swimming and apnea swimming were performed with the same velocity (see Figure 1). The velocity of diving was assessed beforehand on the day prior to the test execution (since diving velocity is usually slower than swimming).

The test of the 100 m Monofin was performed as an official competition discipline, as it is ruled by the international freediving federation CMAS. On the other hand, 100 m with respiration was conducted in the same manner, lacking the whole-body immersion in water. The respiration was allowed through the snorkel. Following that, the pressure at the dry land was at 1 ATA, with the depth freediving it was increasing by 1 ATA every 10 m of the dive [25]. Following this, and because of the nature of pool diving, the athletes experience approximately 1.1 ATA at the depth at which they dive. To be precise, athletes try to stay at a constant depth of 1 m during the whole dive in the pool. Additionally, the depth at which divers experience changes is 45–55 m (4.5–5.5 ATA) [26]. Hence, the influence of pressure on any physiological parameters can be neglected.

### 2.4. Statistical Analyses

Descriptive statistics were measured to assess the arithmetic means and standard deviations (SDs) of all measured variables and are shown in figures as such. The K–S (Kolmogorov–Smirnov) test for normality was used to determine the normal distribution of the data. To determine the differences between the groups of participants, a *t*-test was used to determine the differences in anthropometric indices. Furthermore, to observe the differences between baseline, respiration, and apnea values in velocity, SpO_2_ and lactate values repeated measures ANOVA with Fischer LSD test was used. Following that, the two-factor ANOVA analysis (group (respiration/apnea) and group (intermediate/elite level)) was performed to determine the possible differences between groups in both conditions. Additionally, the differences among the measurements were calculated by the magnitude-based Cohen’s effect size (ES) statistic, with modified qualitative descriptors (trivial ES: <0.2; small ES: 0.21–0.60; moderate ES: 0.61–1.20; large ES: 1.21–1.99; very large ES: >2.0) [27].

Statistica ver. 13.0 (Dell Inc., Austin, TX, USA) and were used for the analyses, and a level of 95% (*p* < 0.05) was applied.

## 3. Results

Table 1 demonstrates differences between intermediate and elite freedivers in age, training age, body height, and body weight. The results present no significant differences between groups in general variables.

**Figure 2:**
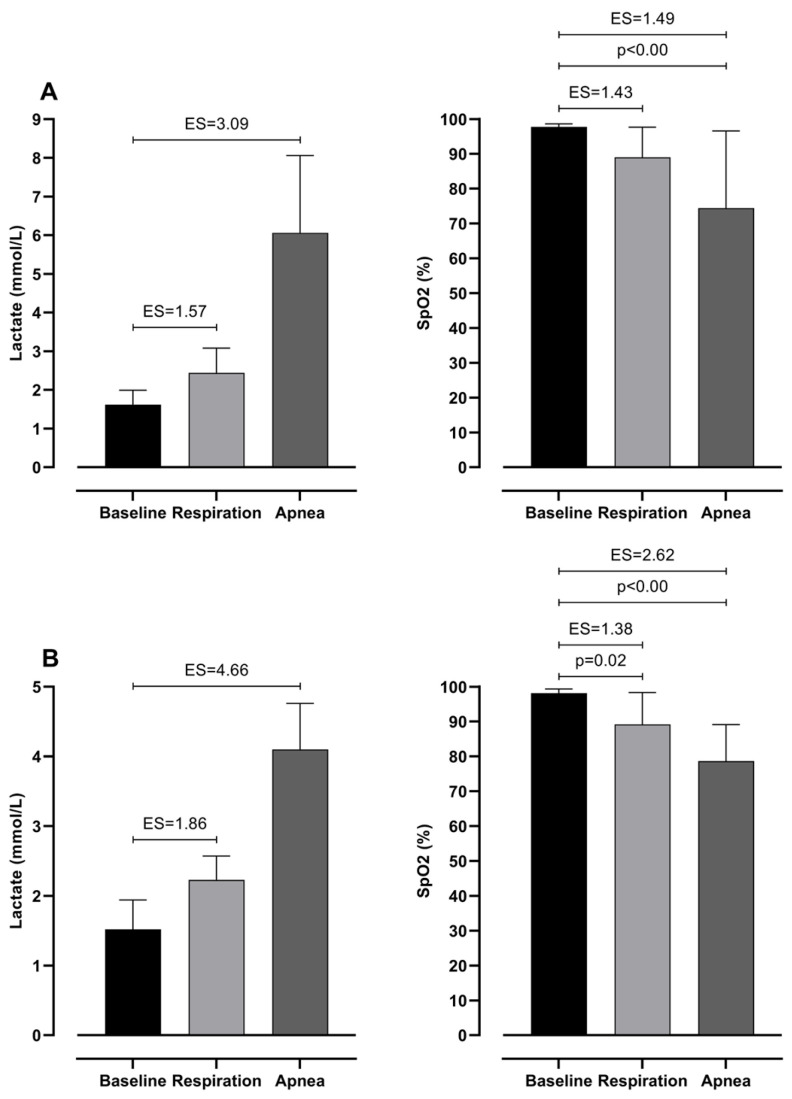
Differences between baseline, respiration, and apnea in Lactates and SpO_2_ with effect sizes (ES), for intermediate (**A**) and elite (**B**) groups.

Repeated measures ANOVA revealed possible differences in both groups for all variables among baseline, respiration, and apnea values (see Table 2). Post hoc analysis of differences between baseline, respiration, and apnea in lactate levels and SpO_2_ can be seen in Figure 2. Even though lactate values showed no significant differences between baseline and test values, a very large effect size is observed. Furthermore, SpO_2_ shows the highest values during the baseline and very large ES difference with respiration and apnea in both groups.

**Figure 3:**
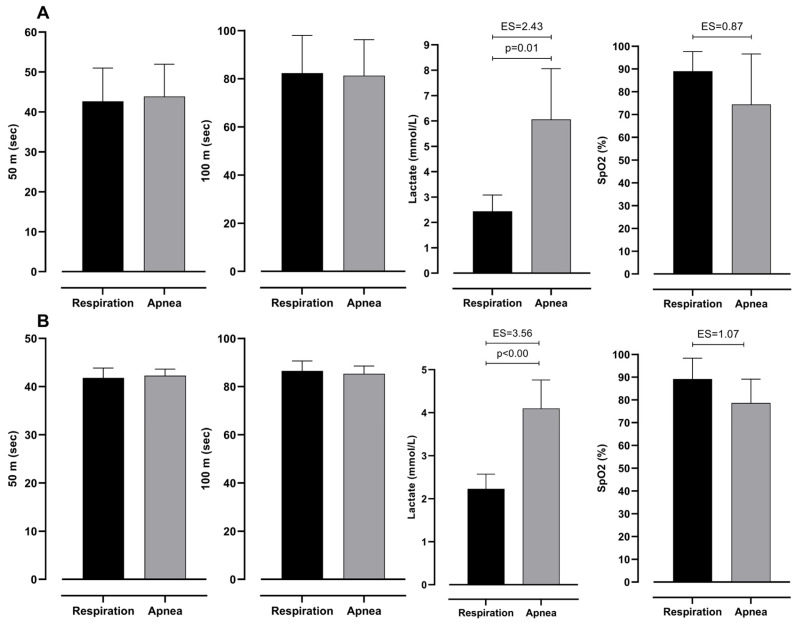
Differences between respiration and apnea in observed variables with effect sizes (ES), for intermediate (**A**) and elite (**B**) groups.

Table 3 presents two-factor ANOVA, which revealed possible differences between groups (elite and moderate) of BHD and type (respiration and apnea) of test. However, it can be seen that there is no significant interaction between them. This shows that both groups have differences in the same variables. Furthermore, lactate levels differ significantly between 100 m dives with respiration and during apnea. In particular, divers perceive significantly smaller levels of lactates during dives with respiration than during apnea. Additionally, SpO_2_ levels do not differ significantly; however, ES shows an intermediate effect between respiration and apnea dive. On the other hand, 50 m and 100 m times do not differ significantly (see Figure 3).

**Figure 4:**
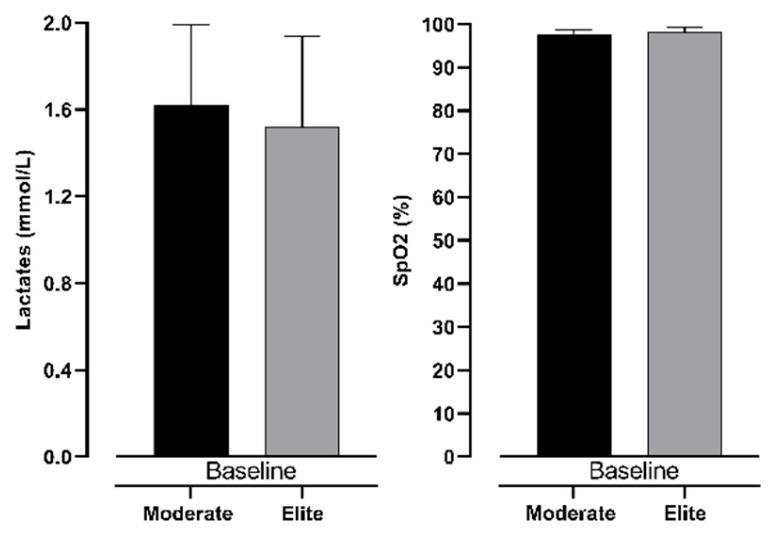
Differences between intermediate and elite freedivers in lactate levels and SpO_2_ in baseline measurement.

**Figure 5:**
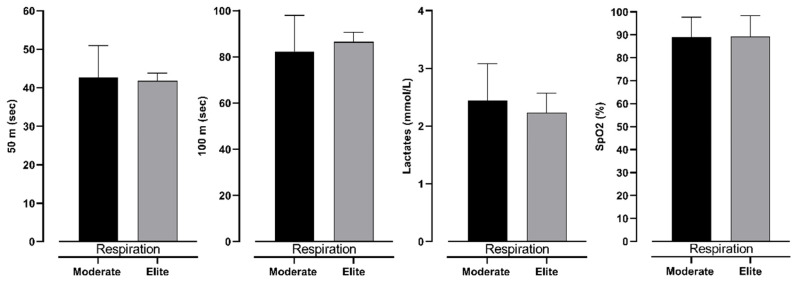
Differences between intermediate and elite freedivers in 50 m, 100 m, lactate levels, and SpO_2_ in respiration measurement, with effect size values (ES).

**Figure 6:**
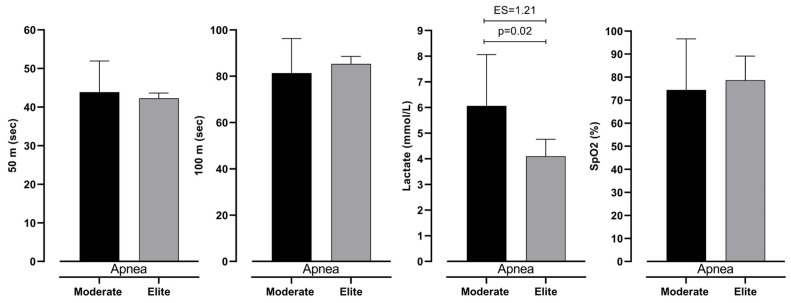
Differences between intermediate and elite freedivers in 50 m, 100 m, lactate levels, and SpO_2_ in Apnea measurement, with effect size values (ES).

The differences in measured variables in all three measurements are presented in Figure 4 and Figure 5. It can be noted that the only significant difference between groups of participants is seen in lactate accumulation (see Figure 6). Other variables did not show significance between groups with trivial ES values.

## 4. Discussion

To the best of our knowledge, this is one of the first studies that has dealt with the anaerobic metabolism response in a specific diving environment of BHD [21]. The results of our study showed a few important findings: (i) 100 m monofin apnea induced higher blood lactates and smaller SpO_2_ values in both groups; (ii) both apnea and respiration 100 m monofin induced changes in blood lactates and SpO_2_; (iii) intermediate BHD produce higher blood lactate levels during 100 m monofin apnea, but not during respiration.

When observing the physiological response to 100 m monofin apnea it can be noted that participants experience significantly higher blood lactate levels than during respiration swimming. Similarly, SpO_2_ levels show lower levels when comparing apnea and respiration tests. In particular, highest values are observed during the baseline (intermediate, 97.78 ± 0.83%; elite, 98.17 ± 1.17%) and moderate in respiration (intermediate, 89.00 ± 8.66%; elite, 89.17 ± 9.17%) and lowest during apnea (intermediate, 74.44 ± 22.15%; elite, 78.67 ± 10.46%). Such findings implicate the influence of DR in anaerobic metabolism increase during apnea. Previous studies demonstrated similar findings, where the DR is highly initiated during apnea, dynamic and static [28,29]. Such a response decreases oxygen delivery to peripheral tissues and redirects blood flow toward vital organs [30,31]. Therefore, lower muscle blood perfusion during DA affects the increase in anaerobic metabolism for energy delivery [32].

Following that, the increase in blood lactate levels implicates a higher demand for anaerobic metabolism in divers in this study. Such demand is necessary for the retention of muscle contraction since divers need to propel themselves during dynamic dives. Additionally, the velocity of swimming in both respiration and apnea swimming showed the same results. Therefore, the only reason for the lower SpO_2_ and higher lactate values is the hypoxic conditions and belonging physiological response in which athletes performed. In our study, intermediate freedivers tend to perceive significantly higher values (6.06 ± 2.00 mmol/L) than elite divers in apnea diving (4.10 ± 0.66 mmol/L). Previously, it was defined that non-apneists perceive DR faster than BHD, mainly because of the oxygen consumption economy (rate of oxygen use during BHD) [33]. Apart from that, BHD has a more pronounced DR than the general population during physical activity [13,33]. The authors imply that despite the characteristics of DR, which occur before the energy deficit [33,34], consumption dynamics of anaerobic reserves manage further regulation of DR. One of the factors for the management of DR is autonomic nervous system response to hypoxia. According to Breskovic, Uglesic, Zubin, Kuch, Kraljevic, Zanchi, Ljubkovic, Sieber, and Dujic [30], voluntary breath holds result in an increase in muscle sympathetic nerve activity (MSNA) and its magnitude is connected to the depth and duration of hypoxia. Heusser et al. have reported that during maximal static apnea, MSNA is increased for 7–8 fold from the baseline values, indicating supramaximal physiological response [4]. Furthermore, we have found that sympathetic postganglionic neurons are recruited based on the size principle; the recruitment of larger, otherwise silent, neurons accounted for approximately 74% of the increase in detected action potentials across MSNA burst sizes [35]. Also, the energy expenditure differences in trained and untrained apneists are present during rest. Such differences multiply during physical activity and lead to higher DR [36,37]. Therefore, the link between DR and training adaptations could be explained by higher energy loss or oxygen consumption during apnea.

The results also imply an increase in anaerobic metabolism during both respiration and apnea swimming. The magnitude of the increase is defined by the energy needs of muscle, state of aerobic reserves, and individual abilities to activate sympathetic and parasympathetic stimulus in terms of DR (e.g., heart rate, cardiac output, and peripheral vasoconstriction). Since DR is a connection between simultaneous sympathetic and parasympathetic coactivation, it could present as an important variable to performance. Also, early DR activation (before energy consumption occurred), presents conservation of new arterial blood received by the splenic contractions in apnea [38,39]. However, the response of the body to protect vital organs will still lead to a change in the metabolism of energy inside muscles. Therefore, higher muscle work will lead to higher oxygen demand and higher sympathetic activity. Hence the accumulation of blood lactate will be present regarding DR. Also, Schagatay [14] reported that even during static apnea, BHD lactates are increased up to 5 mmol/L. This leads to the conclusion that other tissues, more sensitive to limited perfusion, consume oxygen reserves and leave muscles only with anaerobic energy. Brain perfusion was only increased during voluntary pulmonary hyperinflation measured with magnetic resonance imaging, whereas liver perfusion was maintained; thus under this condition of CO, myocardial and skeletal muscle perfusion are reduced [23].

Previous studies defined DR manifestation as a response to functional-energetic metabolism in high anaerobic environments in both static and dynamic apnea [8,12,13,14,34]. In regard to these findings, it can be assumed that elite BHD had lower activity of anaerobic glycolysis than intermediate. This assumption can be seen in the results of lactate accumulation, which is lower in the elite group. Hence, they did not reach the need for change from aerobic to anaerobic metabolism. Additionally, since higher anaerobic abilities are connected to higher buffering capacities of muscle cells [40,41], the anaerobic metabolism response may be influenced by the higher buffering capacity of elite divers. According to Vinetti et al. [42], phosphocreatine (PCr) system contribution showed high involvement in dynamic apnea. The authors stated that such results are connected to PCr hydrolysis, which buffers one H+, making an increased reliance on EPCr, applying not only to O_2_ sparing, but also to pH control. Hence, a highly developed PCr system in elite divers would lead to lower lactate accumulation. Additionally, elite divers most probably have more economic movement patterns, which leads to the lower discharge of energy reserves. With that, their superior technique leads to the inclusion of a lower number of muscle fibers and better diffusion of lactic acid between active and passive muscles.

### 4.1. Limitations and Strengths

This study is one of the first that explores both aerobic and anaerobic metabolism during breath-hold diving. Also, the differences between monofin swimming with and without respiration could present valuable data for athletes and coaches in freediving. Following that, one this study’s main strengths is its sample of participants, which included national team athletes and national and world record holders, therefore presenting a highly representative sample. Moreover, the athletes were tested during pool diving, which is considered an ecologically valid environment. However, the limitations of this study could be seen in the small number of variables measured.

### 4.2. Future Research Suggestions

Following the limitations of this study, future research should be focused on more detailed analysis of diving response with higher number of physiological responses (heart rate, arterial blood sampling, diaphragm movements). Also, the study should be conducted during deep diving, since depth presents even more physiological stress on athletes (pressure). There could be a possibility of testing the control group of non-divers to fully explore the adaptations to which athletes are exposed.

## 5. Conclusions

This study provides valuable insights into monofin diving. It can be noted that apnea swimming is higher in the exhaustion of both aerobic and anaerobic reserves in freedivers. The 100m monofin apnea induced higher blood lactates and lower SpO_2_ levels in both groups. Compared to the elite group, the intermediate BHD produced higher blood lactate levels, which were not observed during respiration. 

The difference between participant groups may be due to DR, training adaptations, or higher energy loss and oxygen consumption during apnea. Elite divers’ efficient movement conserves energy better. Additionally, it can be assumed that longer exposure to training in elite BHD is important for developing better capacities and improving anaerobic capacities. Also, arrhythmic events post-exercise might distinguish the two categories of BHD; hence, future studies should employ 24-h EKG monitoring. Since long-term training exposure shows such effects on BHD (improving lactate tolerance, buffering capacity, apneic training) could be useful for tactical athletes for the improvement of physiological adaptations. These results may help practitioners to improve their breath-hold abilities by engaging in more anaerobic training regimes and apnea training.

## Figures and Tables

**Table 1:** Descriptive parameters and differences between intermediate and elite freedivers in age, training age, and anthropometric indices.

Variables	Intermediate (N = 9)		Elite (N = 6)		*t*	*p*
	Mean	SD	Mean	SD		
Age (years)	25.44	2.74	26.50	3.45	−0.66	0.52
Training age (years)	2.89	1.76	4.33	1.86	−1.52	0.15
Body height (cm)	181.78	8.01	177.87	10.49	0.82	0.43
Body weight (kg)	79.68	9.43	69.30	13.34	1.77	0.10

SD, standard deviation; *t*, test value of *t*-test; *p*, level of significance.

**Table 2:** Repeated measures ANOVA analysis among baseline, respiration, and apnea values of lactate and SpO_2_ in elite and moderate BHD.

Effect	SS	df	MS	F-Value	*p*-Value
	Elite				
Lactate (mmol/L)	21.34	2	10.67	39.19	0.00 *
SpO_2_ (%)	1143.00	2	571.50	7.65	0.01 *
	Moderate				
Lactate (mmol/L)	100.11	2	50.06	36.37	0.00 *
SpO_2_ (%)	2500.07	2	1250.04	5.87	0.01 *

SS, sum of squares; df, degrees of freedom; MS, means of squares; F-value, test value of ANOVA; *p*-value, level of significance set at *p* < 0.05; *, significant value.

**Table 3:** Two-factor ANOVA analysis between group, type, and group * type interaction in all measured variables.

Effect	Wilks Lambda	F-Value	Effect (df)	Error (df)	*p*
Intercept	0.01	705.62	4	23	0.00 *
Group	0.64	3.27	4	23	0.03 *
Type	0.27	15.53	4	23	0.00 *
Group * type	0.79	1.54	4	23	0.23

Group, elite, or moderate BHD; type, respiration or apnea; F-value, test value of ANOVA; df, degrees of freedom; *p*-value, level of significance set at *p* < 0.05; *, significant value.

## Data Availability

All data is contained within manuscript body.
